# Cognition and objective sleep quality in post-COVID-19 patients

**DOI:** 10.3389/fpsyg.2025.1418602

**Published:** 2025-02-03

**Authors:** Anna Carnes-Vendrell, Gerard Piñol-Ripoll, Adriano Targa, Nuria Tahan, Mar Ariza, Neus Cano, Barbara Segura, Carme Junque, Javier Béjar, Cristian Barrue, Nautilus Project Collaborative Group, Linh Viet Nguyen, Maite Garolera

**Affiliations:** ^1^Cognitive Disorders Unit, Cognition and Behaviour Study Group, Hospital Universitari Santa Maria, Lleida, Spain; ^2^Translational Research in Respiratory Medicine, Hospital Universitari Arnau de Vilanova-Santa Maria, IRBLleida, Lleida, Spain; ^3^Clinical Research Group for Brain, Cognition and Behaviour, Consorci Sanitari de Terrassa, Terrassa, Spain; ^4^Departament de Ciències Bàsiques, Universitat Internacional de Catalunya (UIC), Barcelona, Spain; ^5^Medical Psychology Unit, Department of Medicine, Universitat de Barcelona, Barcelona, Spain; ^6^Institute of Neurosciences, University of Barcelona, Barcelona, Spain; ^7^Institut d’Investigacions Biomèdiques August Pi i Sunyer (IDIBAPS), Barcelona, Spain; ^8^Centro de Investigación Biomédica en Red sobre Enfermedades Neurodegenerativas (CIBERNED), Barcelona, Spain; ^9^Faculty of Informatics of Barcelona (FIB), Polytechnic University of Catalonia, Barcelona, Spain; ^10^Neuropsychology Unit, Consorci Sanitari de Terrassa, Terrassa, Spain

**Keywords:** actigraphy, cognition, post-COVID-19 condition, sleep efficiency, sleep quality, total sleep time

## Abstract

In the current study, we aimed (i) to evaluate sleep quality via wrist actigraphy monitoring of nonhospitalized and hospitalized post-COVID-19 condition (PCC) participants; (ii) to correlate actigraphy measures with subjective measures of sleep quality, such as the Pittsburgh Sleep Quality Index (PSQI); and (iii) to investigate whether total sleep time or sleep efficiency could affect PCC cognitive performance. We included 49 individuals with PCC from the NAUTILUS Project (NCT05307549 and NCT05307575) who were monitored for 1 week via actigraphy and who were also assessed with a comprehensive neuropsychological battery and the PSQI. We found that there were significant differences between nonhospitalized PCCs and hospitalized PCCs in the number of awakenings. We also found a correlation between the total sleep time of both measures (actigraphy and PSQI), but we did not observe correlations between objective and subjective parameters of latency and sleep efficiency. Regarding cognition and actigraphy measures, there was a trend of statistical significance in the performance of immediate visual memory, attention span and social cognition according to sleep efficiency. In conclusion, results indicate that although the PSQI provides clinically relevant indicators of sleep, there are divergent results between self-reported and objective sleep measures (actigraphy). Furthermore, we found a tendency toward statistical significance in cognitive performance in PCC participants according to their sleep efficiency which could indicate that is more important for cognitive function of post-COVID-19 patients than total sleep time.

## 1 Introduction

Sleep quality has been widely studied in post-COVID-19 condition (PCC) participants. It has been proven that sleep disturbances are among the most prevalent symptoms in PCC patients, along with cognitive (Ziauddeen et al., 2022; García-Sánchez et al., 2022; Delgado-Alonso et al., 2022; Matias-Guiu et al., 2022; Davis et al., 2021; Guo et al., 2022; Ariza et al., 2022) and emotional alterations (Bourmistrova et al., 2022; Vindegaard and Benros, 2020). In fact, many studies have shown that there is a reduction in sleep quality both in hospitalized and nonhospitalized patients (Akıncı and Melek Başar, 2021; Samushiya et al., 2022; Al-Ameri et al., 2022; Malik et al., 2022; Karimi et al., 2022). However, according to our previous study, there is no significant difference in the severity of the disease (Carnes-Vendrell et al., 2024). However, most of the studies included only subjective sleep quality measures, such as the Pittsburgh Sleep Quality Index (PSQI), because of its ease of administration and due to the fact that it requires less time.

With objective measures such as wrist-actigraphy, it is possible to describe the sleep patterns of the participants in a more realistic manner. Thus, it allows for simultaneous ecological assessment of sleep with noninvasive monitoring, as opposed to polysomnography, which usually requires an overnight stay in the hospital and then an expensive sleep evaluation. According to the Academy of Sleep Medicine, the actigraphy provide useful information and it may be cost-effective method for assessing specific sleep disorders (Ancoli-Israel et al., 2003).

To date, several studies have been published using actigraphy methods to analyze sleep patterns and circadian rhythms during the COVID-19 lockdown. In fact, a systematic review was published in 2023 to summarize the previous literature in this field (Ferreira-Souza et al., 2023). They concluded that actigraphy should be included as part of the sleep hygiene strategy, as it may be the best instrument for obtaining data about sleep patterns. However, only one of the included 15 studies involved COVID-19 patients, and the remaining studies involved the general population (medical staff, children, and students, among others).

There is a large amount of evidence that sleep plays a fundamental role in the regulation of emotions and adequate cognitive functioning. In fact, a lack of adequate sleep is a major source of many harmful diseases related to the heart, the brain, psychological changes, high blood pressure, diabetes, and/or weight gain (Belal et al., 2020; Salehinejad et al., 2022). Previous findings in older adults support the idea that reduced sleep quality can contribute to poor cognitive performance (Lim et al., 2013; Naismith et al., 2010; Lambiase et al., 2014; Blackwell et al., 2006; Blackwell et al., 2011; Yaffe et al., 2014). However, to our knowledge, the relationship between sleep quality and cognition in the PCC has not been studied.

Therefore, in the current study, we aimed (i) to evaluate sleep quality through wrist actigraphy monitoring of nonhospitalized and hospitalized post-COVID-19 condition (PCC) participants; (ii) to correlate actigraphy measures with subjective measures of sleep quality, such as the Pittsburgh Sleep Quality Index (PSQI); and (iii) to investigate whether total sleep time or sleep efficiency could affect PCC participants’ cognitive performance.

## 2 Methods

### 2.1 Participants

We included 49 participants from the Nautilus Project (ClincalTrials.gov IDs: NCT05307549 and NCT05307575), 35 of whom were nonhospitalized, which means that only showed mild COVID-19 symptoms in the acute phase and stayed at home, and 14 of whom were hospitalized (presenting moderate to severe symptoms of the disease). The samples were collected at Hospital Universitari Santa Maria (Lleida, SPAIN) and Hospital Universitari Arnau de Vilanova (Lleida, SPAIN).

The inclusion criteria for the PCC group were a confirmed diagnosis of COVID-19 according to the WHO criteria with signs and symptoms of the disease observed during the acute phase, a period of at least 12 weeks after infection, and age between 18 and 65 years. The exclusion criteria were an established diagnosis of a psychiatric disorder, neurological disorder, neurodevelopmental disorder, or systemic pathology known to cause cognitive deficits before COVID-19 infection and motor or sensory alterations that could interfere with the neuropsychological assessment.

### 2.2 Procedure

Participants were recruited at the Cognitive Disorder Unit of Hospital Universitari Santa Maria (Lleida, SPAIN) and Hospital Universitari Arnau de Vilanova (Lleida, SPAIN). We obtained written informed consent from all of the participants before inclusion. We collected data on sociodemographic characteristics, previous comorbidities and COVID-19 symptoms in the first session. At the second visit, a neuropsychological assessment was performed. Different cognitive domains were assessed with an extensive and comprehensive neuropsychological battery that was described in a previous study (Carnes-Vendrell et al., 2024). All of the evaluations were performed by trained neuropsychologists. The participants were also asked to complete different questionnaires, including the Pittsburgh Sleep Quality Index (PSQI), which allowed us to assess sleep quality. Finally, the participants were monitored for 7 days with a wrist-mounted actigraph (Actiwatch 2, Philips Respironics, Murrysville, PA). We obtained the following variables: total sleep time (hours), time in bed (hours), sleep efficiency (%, defined as the ratio between total sleep time and the time spent in bed), sleep latency (minutes, defined as the time spent awake until the first sleep episode), time spent awake after sleep onset (WASO (minutes), and number of awakenings).

The participants’ anonymity and confidentiality were guaranteed. The Scientific Ethics Committee of the Hospital Universitari Arnau de Vilanova approved both the study and the consent procedure (CEIC 2384), as did the Drug Research Ethics Committee (CEIm) of Consorci Sanitari de Terrassa (CEIm code: 02–20–107-070) and the Ethics Committee of the University of Barcelona (IRB00003099). Additionally, the investigation was conducted in accordance with the latest version of the Declaration of Helsinki.

### 2.3 Statistical analysis

Descriptive analyses were performed on PCC patients (nonhospitalized vs. hospitalized). For categorical variables, frequencies and percentages were obtained, and for quantitative variables, the means and standard deviations or medians and interquartile ranges (IQRs) were obtained. For the sociodemographic and clinical profiles, quantitative variables were compared between severity groups by using Student’s *t* test or the Mann–Whitney test, according to normality (verified by using the Shapiro–Wilk test). For categorical variables, groups were compared by using Pearson’s chi-squared tests (or the Fisher’s exact test, if applicable).

To analyze the relationship between the PSQI score and the actigraphy parameters (sleep latency, sleep efficiency and total sleep time), Spearman’s rho correlations were used, given the nonnormality of the parameters.

To analyze the relationships between cognitive parameters and actigraphy parameters (total sleep time and sleep efficiency), nonparametric Pearson’s chi-squared tests were used (or the Fisher’s exact test was applied, if appropriate). Both total sleep time and sleep efficiency were dichotomized according to the median, and cognition variables were converted into dichotomous variables depending on whether the result indicated cognitive impairment (−1 standard deviation from the mean).

The statistical significance level that was used in the analyses was 5% (*α* = 0.05). All of the analyses were performed with IBM SPSS statistics 26.

## 3 Results

Of the 49 PCC patients, 35 were not hospitalized, and the mean (M) age was 48.80 years (standard deviation [SD]: 8.75); 14 were hospitalized, with a mean age of 55.14 years (SD: 5.86), and this difference was significant (*p* = 0.016). In the nonhospitalized-PCC group, the majority of participants were female (77.11%) and had more years of education (M: 14.79; SD: 2.33) (*p* = 0.042). Participants in the hospitalized PCC group had more comorbidities, such as high blood pressure (42.9%), dyslipidaemia (35.7%), and obesity (42.9%). Table 1 shows the clinical and sociodemographic characteristics of the sample.

**Table 1:** Clinical and sociodemographic characteristics of the sample.

	Non-hospitalitzed PCC	Hospitalized PCC	*p value*
	*n* = 35	*n* = 14	
Age (years) (SD)	48.80 (8.75)	55.14 (5.86)	0.016*
Female (%)	77.11%	42.9%	0.021*
Years of education (SD)	14.79 (2.33)	13.14 (2.85)	0.042*
Days since COVID-19 (SD)	453.91 (287.82)	363.21 (134.52)	0.141
MoCA (SD)	25.79 (2.58)	25.43 (2.38)	0.65
BMI (SD)	26.56 (5.34)	28.66 (3.97)	0.192
Tobacco smoking (%)	5.9%	14.3%	0.338
Alcohol consumption (%)	50.0%	21.4%	0.068
Previous comorbidities			
Heart disease (%)	0.0%	0.0%	-
Respiratory disease (%)	14.7%	0.0%	0.13
Chronic kidney disease (%)	0.0%	0.0%	-
High blood pressure (%)	11.8%	42.9%	0.016*
Dyslipidemia (%)	14.7%	35.7%	0.103
Diabetes mellitus (%)	2.9%	7.1%	0.508
Obesity (%)	17.6%	42.9%	0.067
Chronic liver disease (%)	0.0%	0.0%	-
Chronic pain (%)	2.9%	0.0%	0.517
Quality of sleep			
PSQI total score	8.70 (3.20)	9.50 (4.50)	0.803
Poor quality of sleep (>5)	81.8%	78.6%	0.796
Actigraphy variables			
Total sleep time, hours (Mdn, IQR)	6.73 (5.93–7.48)	6.78 (6.15–7.27)	0.851
Time in bed, hours (Mdn, IQR)	8.27 (7.77–8.72)	7.96 (7.67–8.60)	0.432
Sleep efficiency, % (Mdn, IQR)	84.99 (77.69–88.24)	85.62 (81.21–87.45)	0.298
Sleep latency, minutes (Mdn, IQR)	14.00 (9.00–22.00)	15.00 (12.00–23.00)	0.565
WASO, minutes (Mdn, IQR)	45.00 (27.00–61.00)	44.00 (25.00–52.00)	0.507
Number of awakenings (Mdn, IQR)	36.33 (28.71–43.86)	25.84 (18.67–34.71)	0.008*

Unless otherwise specified, results are presented as mean (standard deviation).

Mdn, median; IQR, interquartile range.

Bold values mean level of statistical significance = *p < 0.05, **p < 0.01, ***p < 0.001.

PCC, Post-COVID-19 Condition; MoCA, Montreal Cognitive Assessment; BMI, Body Mass Index; WASO, wake after sleep onset.

In terms of sleep quality, the hospitalized PCC participants obtained a mean PSQI total score of 9.50 (SD: 4.50), and the nonhospitalized PCC participants obtained a mean score of 8.70 (SD: 3.20); however, this difference was not significant (*p* = 0.803). The nonhospitalized-PCC group had a percentage of participants with a score above 5 on the PSQI which indicates poorer sleep quality (81.8% vs. 78.6%, respectively), with no significant differences between the groups (*p* = 0.796) (Table 1).

According to the actigraphy variables, there were only significant differences in the number of awakenings (*p* = 0.008) between nonhospitalized PCCs (M: 36.33; IR: 28.71–43.86) and hospitalized PCCs (M: 25.84; IR: 18.67–34.71) (Table 1). We did not observe significant differences in the remaining actigraphy variables, such as sleep latency and efficiency, time in bed or total sleep time, between the nonhospitalized and hospitalized PCC patients.

Regarding the correlation between subjective and objective measures of sleep variables, we evaluated the correlation of sleep latency, sleep efficiency and total sleep time according to the PSQI and actigraphy. We found a correlation between total sleep time and both measures (*p* = 0.009; Rho Spearman: −0.447) in the nonhospitalized PCC group (Table 2). This negative correlation should be interpreted considering that the higher the PSQI score (which indicates worse sleep quality), the fewer the hours of total sleep time according to the actigraph.

**Table 2:** Correlations between objective and subjectives sleep measures.

	Total PCC sample	Non-hospitalized PCC	Hospitalized PCC
	*n* = 49	*n* = 35	*n* = 14
Sleep latency	−0.074	−0.034	−0.208
	*p* = 0.622	*p* = 0.850	*p* = 0.475
Sleep efficiency	−0.013	−0.009	0.017
	*p* = 0.930	*p* = 0.960	*p* = 0.955
Total sleep time	**−0.396**	**−0.447**	−0.370
	*p* = 0.006**	*p* = 0.009**	*p* = 0.193

Spearman’s Rho correlation.

Bold values mean level of statistical significance = *p < 0.05, **p < 0.01, ***p < 0.001.

Finally, we evaluated the cognition parameters according to the total sleep time and sleep efficiency of the whole sample (Table 3). We did not observe significant differences in cognition between groups in terms of total sleep time or sleep efficiency. There was a trend toward statistical significance in the performance of immediate visual memory (*p* = 0.054), attention span (*p* = 0.056) and social cognition (*p* = 0.062) according to sleep efficiency; specifically, a lower sleep efficiency corresponded to a worsened cognitive performance.

**Table 3:** Group comparison of cognitive performance in PCC participants according to total sleep time and sleep efficiency.

			Total sleep time	Sleep efficiency
Memory	RAVLT total score	Chi-square	1.742	0.17
		Sig.	0.187	0.68
	RAVLT immediate recall	Chi-square	1.38	0.294
		Sig.	0.24	0.588
	RAVLT delayed recall	Chi-square	1.557	0.34
		Sig.	0.212	0.56
	ROCF immediate recall	Chi-square	3.714	3.714
		Sig.	0.099	0.054
	ROCF delayed recall	Chi-square	0.903	0.903
		Sig.	0.342	0.342
Attention and processing speed	Digit span forward	Chi-square	1.557	3.657
		Sig.	0.212	0.056
	Digit span backward	Chi-square	1.002	1.002
		Sig.	0.609	0.609
	Digit Symbol	Chi-square	2.002	2.002
		Sig.	0.490	0.490
	TMT A	Chi-square	0.903	0.903
		Sig.	0.342	0.342
	TMT B	Chi-square	0.003	0.003
		Sig.	1.000	1.000
Executive functions	Stroop color word	Chi-square	0.523	0.294
		Sig.	0.47	0.588
	Verbal fluency_P	Chi-square	0.405	0.005
		Sig.	0.725	1.000
	Verbal fluency_M	Chi-square	3.329	3.329
		Sig.	0.110	0.068
	Verbal fluency_R	Chi-square	0.504	0.699
		Sig.	0.702	0.375
Language	Semantic fluency	Chi-square	0.091	0.091
		Sig.	1.000	1.000
	BNT	Chi-square	0.699	0.004
		Sig.	0.463	1.000
Praxis	ROCF copy trial	Chi-square	0.008	0.294
		Sig.	0.928	0.588
Social cognition	Eye test	Chi-square	0.525	3.496
		Sig.	0.469	0.062

Pearson’s Chi square results.

Level of statistical significance = *p < 0.05, **p < 0.01, ***p < 0.001.

PCC, Post-COVID-19 Condition; RAVLT, Rey Auditory Verbal Learning Test; ROCF, Rey–Osterrieth Complex Figure Test; TMT, Trail Making Test; BNT, Boston Naming Test.

## 4 Discussion

Our study demonstrated significant differences only in the number of awakenings between nonhospitalized and hospitalized PCC patients. Therefore, for the remaining actigraph parameters, PCC participants had the same results regardless of the severity of PCC. When we correlated the wrist-actigraphy results with self-reported variables (PSQI), we found a significant correlation with total sleep time. Finally, when we analyzed the relationships between cognitive performance and total sleep time and sleep efficiency, we found a tendency toward statistical significance in visual memory, attention and social cognition according to sleep efficiency.

Some previous research has shown similar results in terms of subjective and objective measurements of sleep quality. Different studies that were performed only with nonhospitalized PCC participants have obtained results similar to our results for the actigraph parameters (Reid et al., 2024; Henríquez-Beltrán et al., 2022). In addition, our results are comparable to those of previous studies that included only samples of hospitalized PCC patients (Benítez et al., 2022; Targa et al., 2022; Jackson et al., 2023; Henríquez-Beltrán et al., 2022). To our knowledge, there is only one study that included nonhospitalized and hospitalized PCC participants and compared the actigraphy parameters between them. In that study, the authors did not observe a significant difference among PCC participants according to the severity of the disease (Tański et al., 2024). Thus, our results confirm the absence of a relationship between the severity of PCC and actigraphy parameters.

Most of the previous studies that also used wrist-actigraphy and the PSQI did not perform correlation analyses. We demonstrated a correlation between objective and subjective measurements in terms of total sleep time. Tański et al. (2024) also found the same correlation, but they used the Epworth Sleepiness Scale (ESS), which is used to assess daytime sleepiness. Their results also demonstrated correlations between the ESS score and total time in bed and wakefulness after sleep onset. Given the absence of previous studies that analyze the correlation between objective and subjective sleep data, it is not possible to draw any conclusions other than those described. Perhaps the fact that self-reported and subjective measures do not enjoy the same reliability as objective ones can make that many researchers do not consider analyzing this correlation.

Finally, we investigated whether cognitive performance is related to actigraphy parameters such as total sleep time and sleep efficiency. Based on previous studies that demonstrated the importance of good-quality sleep for good cognitive functioning, we analyzed these two variables. Unexpectedly, we did not observe significant results. However, this may be due to the small sample size because we did observe a tendency toward statistical significance in visual memory, attention and social cognition according to sleep efficiency. To our knowledge, only one previous recent study has analyzed cognitive performance and sleep quality in COVID-19 patients. The authors also found that lower sleep efficiency was associated with lower attention and processing speed (Reid et al., 2024). In studies focusing on the role of sleep for cognition but not in COVID-19 population, it is said that sleep is crucial for cognition, specially in elderly people. Sleep fragmentation and absence of deep sleep has been associated with worse cognitive function in older people (Lim et al., 2013; Naismith et al., 2010; Yaffe et al., 2014; Targa et al., 2021).

The strengths of the study include the use of both objective and self-reported measures of sleep quality, such as the PSQI and wrist-actigraphy. In addition, we performed a comprehensive cognitive assessment, which allowed us to explore the possible effects of sleep quality on all cognitive domains. However, several limitations should be considered. First, our relatively small sample size may have limited our ability to achieve statistically significant results. Second, the absence of a healthy control group prevented us from extending our conclusions to the general population. Finally, we cannot forget that this is a cross-sectional study; thus, a longitudinal follow-up would be necessary to observe the evolution of sleep quality and its alterations in PCC participants to determine how they evolve over time.

Despite the end of the pandemic, COVID-19 is still present in the population, and the disease may have a long-lasting impact on health due to persistent COVID-19 symptoms, such as sleep and emotional disturbances, as well as cognitive impairment. Our results confirm that PCC participants suffer from poor sleep quality, which has been corroborated both with objective and subjective measures, and that this may affect their cognitive performance, especially in memory, attention, and social cognition tasks. Although our study did not show significant results for many variables, as we expected, we believe that it is relevant because of its implications. Therapeutic strategies focused on sleep quality may imply improvements in different areas, such as cognition and psychological processes.

## Data Availability

The raw data supporting the conclusions of this article will be made available by the authors, without undue reservation.

## Group members of Nautilus Project Collaborative Group

Vanesa Arauzo and Jose A. Bernia, Consorci Sanitari de Terrassa (CST), Terrassa, Spain. Marta Balague-Marmaña and Berta Valles-Pauls, Hospital Sant Joan Despí Moisès Broggi, Consorci Sanitari Integral. Jesús Caballero, Hospital Universitari Arnau de Vilanova, Lleida, Spain. Ester Gonzalez-Aguado and Carme Tayó-Juli, Consorci Sanitari Alt Penedès-Garraf, Vilafranca de Penedés, Barcelona, Spain. Eva Forcadell-Ferreres and Silvia Reverte-Vilarroya, Hospital Verge de la Cinta, Tortosa, Tarragona, Spain. Susanna Forné, Fundació Sant Hospital de la Seu d’Urgell, La Seu d’Urgell, Lleida, Spain. Anna Bartes-Plans and Jordina Muñoz-Padros, Consorci Hospitalari de Vic, Vic, Barcelona, Spain. Jose A. Muñoz-Moreno and Anna Prats-Paris, Servei de Malalties Infeccioses, Fundació Lluita contra les Infeccions – Hospital Universitari Germans Trias i Pujol, Badalona, Barcelona, Spain. Inmaculada Rico and Nuria Sabé, Hospital Universitari de Bellvitge, L’Hospitalet de Llobregat, Barcelona, Spain. Marta Almeria and Laura Casas, Hospital Universitari Mútua Terrassa, Terrassa, Barcelona, Spain. Maria José Ciudad and Anna Ferré, Badalona Serveis Assistens, Badalona, Barcelona, Spain. Tamar Garzon and Manuela Lozano, Institut d’Assistència Sanitària, Girona, Spain. Marta Cullell and Sonia Vega, Fundació Salut Empordà, Figueres, Girona, Spain. Sílvia Alsina, Fundació Hospital de Puigcerdà, Puigcerdà, Girona, Spain. Maria J. Maldonado-Belmonte and Susana Vazquez-Rivera, Hospital Universitario Central de la Cruz Roja San José y Santa Adela, Madrid, Spain. Eva Baillès and Sandra Navarro, Servei Andorrà d’Atenció Sanitària (SAAS), Andorra. Ayoze González Hernández, Facultad de Ciencias de la Salud, Universidad Fernando Pessoa Canarias. Yaiza Molina, Clínica Universitaria de Psicología, Facultad de Ciencias de la Salud, Universidad Fernando Pessoa Canarias. Victoria Olive, Occupational Health Care Service, Hospital Clínic Barcelona. Silvia Cañizares, Section of Clinical Psychology of Health, Clinical Institute of Neurosciences, Hospital Clinic of Barcelona. Department of Clinical Psychology and Psychobiology, Universitat de Barcelona.
